# Influence of Spatial Resolution on the Accuracy of Quantitative Myocardial Perfusion in First Pass Stress Perfusion CMR

**DOI:** 10.1002/mrm.25249

**Published:** 2014-05-20

**Authors:** Niloufar Zarinabad, Amedeo Chiribiri, Gilion L T F Hautvast, Marcel Breeuwer, Eike Nagel

**Affiliations:** 1 Division of Imaging Sciences and Biomedical Engineering, King's College London BHF Centre of Excellence, NIHR Biomedical Research Centre and Wellcome Trust and EPSRC Medical Engineering Centre at Guy's and St. Thomas' NHS Foundation Trust, The Rayne InstituteSt. Thomas' Hospital, London, SE1 7EH, UK; 2 Philips Group Innovation—Healthcare IncubatorsEindhoven, The Netherlands; 3 Philips Healthcare, Imaging Systems—MRVeenpluis 4–6, 5680 DA, Best, The Netherlands; 4 Biomedical Engineering, Biomedical Image Analysis, Eindhoven University of TechnologyEindhoven, The Netherlands

**Keywords:** myocardial perfusion quantification, spatial resolution, voxel-wise, perfusion CMR

## Abstract

**Purpose:**

High-resolution myocardial perfusion analysis allows for preserving spatial information with excellent sensitivity for subendocardial ischemia detection. However, it suffers from low signal-to-noise ratio. Commonly, spatial averaging is used to increase signal-to-noise ratio. This bears the risk of losing information about the extent, localization and transmurality of ischemia. This study investigates spatial-averaging effects on perfusion-estimates accuracy.

**Methods:**

Perfusion data were obtained from patients and healthy volunteers. Spatial averaging was performed on voxel-based data in transmural and angular direction to reduce resolution to 50, 20, and 10% of its original value. Fit quality assessment method is used to measure the fraction of modeled information and remaining unmodeled information in the residuals.

**Results:**

Fraction of modeled information decreased in patients as resolution reduced. This decrease was more evident for Fermi and exponential in transmural direction. Fermi and exponential showed significant difference at 50% resolution (Fermi *P* < 0.001, exponential *P* =0.0014). No significant differences were observed for autoregressive-moving-average model (*P* = 0.081). At full resolution, autoregressive-moving-average model has the lowest fraction of residual information (0.3). Differences were observed comparing ischemic regions perfusion-estimates coefficient of variation at transmural and angular direction.

**Conclusion:**

Angular averaging preserves more information compared to transmural averaging. Reducing resolution level below 50% at transmural and 20% at angular direction results in losing information about transmural perfusion differences. Maximum voxel size of 2 × 2 mm^2^ is necessary to avoid loss of physiological information due to spatial averaging. **Magn Reson Med 73:1623–1631, 2015. © 2014 The Authors. Magnetic Resonance in Medicine Published by Wiley Periodicals, Inc. on behalf of International Society of Medicine in Resonance.**

## INTRODUCTION

Dynamic contrast enhanced cardiovascular magnetic resonance (perfusion CMR) is increasingly becoming a routine clinical tool to explore the presence and extent of myocardial ischemia (1–5).

Perfusion CMR permits tracking of temporal variations in contrast agent concentrations and deriving physiological flow parameters within the tissue, such as myocardial blood flow (MBF) estimation using deconvolution methods (6). In addition, the high spatial resolution conferred by CMR permits high-resolution (voxel-wise) myocardial perfusion quantification and allows for the detection of subendocardial perfusion abnormalities. A few recently published studies have demonstrated the potential of quantitative voxel-wise perfusion analysis in improving the clinical diagnostic accuracy (7,8).

Nevertheless, it is imperative to take into account the poor signal-to-noise ratio (SNR) of high-resolution voxel-based data which might result in inaccuracies in quantitative voxel-wise perfusion CMR analysis.

To address the latter issue and obtain higher accuracy, myocardial voxels can be grouped to increase the region of interest (ROI) size (spatial averaging). However, the downside of this approach will be the reduction of spatial resolution and potential information loss on the localization, extent and transmurality of myocardial ischemia.

In this study, we aimed to assess the relationship between the level of spatial resolution of perfusion CMR data and accuracy of perfusion quantification on different methods including Fermi function modeling (9), autoregressive-moving-average (ARMA) model (10), and exponential basis deconvolution (11). In addition, we used a new method developed by Balvay et al. (12) to measure the quality of the fit and the ratio of the modeling error that occurs as a result of low SNR or reduced spatial resolution. This ratio, which is called fraction of residual information (FRI), allows extracting the modeling error from the residuals and is an estimate of the amount unmodeled information remaining in the residuals.

## THEORY

Measured blood,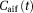, and tissue enhancement data,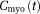, during the first pass of contrast agent in myocardium are related together through the following equation (13):


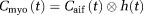
(1)

where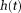 is the tissue impulse response.


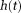
 and consequently tissue characteristics such as perfusion can be estimated from Eq. [1] using deconvolution algorithms such as Fermi function modeling, ARMA and exponential basis deconvolution.

For the estimation of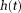, the parameters of these deconvolution models are adjusted to obtain a modeled tissue dynamics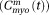 that is as faithful as possible to the observed myocardial curves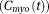, generally based on least-square regression.


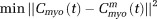
(2)

When the analysis is applied to each ROI (e.g., segment or voxel), it generates maps of estimated MBF.

However, there is no guarantee that the modeled myocardial dynamics will effectively match the observed myocardial curves. In the absence of a reference value available to assess the accuracy of the results (in vivo data), the quality of fit must therefore be determined to avoid erroneous interpretation of MBF.

So far visual analysis and mean square error (MSE) (14,15) are commonly used to verify the quality of fit. These criteria, which are based on measuring the difference between modeled tissue curve and measured data, depend not only on the true quality of fit but also on the level of random noise contained in the dataset.

To prove the above statement, it can be assumed that the observed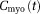 derived from a series of perfusion CMR acquisitions are the sum of a deterministic phenomenon of interest and a random noise θ(*t*):


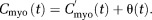
(3)

In practice, deconvolution decomposes these data into the sum of a modeled signal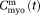 and a residual (*r*):


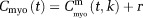
(4)

where


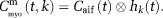



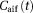
 is the measured arterial input concentration of contrast agent data from left ventricular (LV) and k = {k_1_, …, k_n_} are the n parameters of the model used to represent *h*(*t*). Deconvolution process searches for the parameters of the impulse response (*k*_i_) which minimize the following MSE problem:


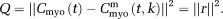
(6)

Using Eq. [3], *Q* can then be expressed as


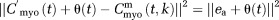
(7)

where *e*_a_ is the true modeling error defined as the difference between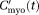 and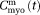:


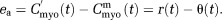
(8)

Equation [7] shows that, the MSE (*Q*) depends on both the true modeling error (*e*_a_) and the noise amplitude (*θ*) and it does not perfectly reflect the quality of fit as it is dependent on noise amplitude. As the modeling error, *e*_a_ and the noise error, *θ*(*t*) cannot be discriminated, the MSE (*Q*) cannot be used to study the relation between quality of fit and resolution level in voxel-wise perfusion CMR.

To discriminate between the fraction of error due to noise and the fraction of modeling error, Balvay et al. (12) have used a quadratic modeling MSE (*Q*′) defined as


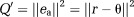
(9)

Here the quality of fit improves as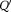 decreases and is perfect when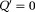.

In an appropriate fit where most of the data's information had been fitted to a model, the modeling error *e*_a_ must be negligible relative to the real tissue curve (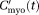).

Balvay et al. (12) used these properties and defined the fraction of modeled information (FMI) to characterize the amount of modeled information:


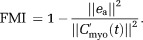
(10)

This criterion represents (*e*_a_) relative to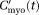 and is not dependent on SNR.

Moreover to represent the nonmodeled information (*e*_a_) relative to the total residual, he intercede the FRI as


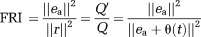
(11)

The FRI ratio is associated with the ability to extract the modeling error from residual which contains the error due to noise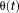. These new fit quality measures will reach their maxima and minima: FMI = 1, FRI = 0.

However, in the case of patient data, the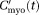 is unknown as we only have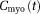 available. Therefore, FRI and FMI must be estimated in presence of noise. Balvay et al. (12) have used a model, based on the properties of the autocorrelation function, to measure an estimate of the *e*_a_ and thus assess the fit quality.

By assuming that *r* = *e*_a_ + *θ*, for each time lag *l*:


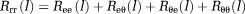


where *R* is the autocorrelation function.

For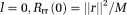 (*M* is the total number of samples).

Assuming *e*_a_ is deterministic and *θ* is white noise:


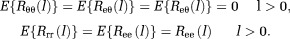


*R*_rr_ is, therefore, an unbiased estimator of *R*_ee_ when *l* > 0.

This will not be true when *l* = 0, as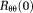, is no longer negligible. However, by *e*_a_ being deterministic,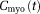,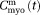 and therefore, *e*_a_ vary moderately with time. As a result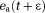 approximates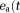) for low values of *l* (i.e., 0 + *ε*) and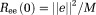 can be approximated using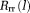 for low values of *l*.

The term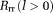 is fitted by a polynomial function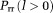, and then *P*_rr_ is extrapolated by continuity to zero to estimate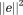. By applying this calculation to the dataset,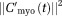 also can be estimated by


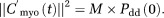


Therefore


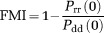
(15)


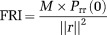
(16)

## METHODS

Perfusion data were analyzed from perfusion CMR scans acquired in seven patients with angina symptoms and definite coronary artery disease on invasive coronary angiography referred for contrast-enhanced first-pass CMR and three healthy volunteers. All participants gave written informed consent for the study, according to the local ethics committee approval.

### Data Acquisition—CMR Protocol

CMR was performed on a 3T system (Achieva TX, Philips Healthcare, Best, The Netherlands) using a 32-channel cardiac phased array receiver coil. Perfusion data were acquired in 3 LV (apical, mid-cavity, and basal) short-axis slices at mid-expiration with a saturation-recovery gradient echo method (repetition time/echo time 3.0 ms/1.0 ms, flip angle 15°, saturation-recovery delay 120 ms, fivefold k-t sensitivity encoding (k-t SENSE) acceleration with 11 training profiles, giving a net acceleration of 3.8-fold, spatial resolution 1.2 × 1.2 × 10 mm^3^). Data were acquired during adenosine-induced hyperemia (140 µg/kg/min) 0.075 mmol/kg of body weight gadolinium (Gd) extracellular contrast agent (gadobutrol, Gadovist®, Bayer, Germany) injected at 4 mL/s followed by a 20-mL saline flush for each perfusion CMR acquisition. Each bolus of gadobutrol was preceded by a diluted prebolus with 10% of the dose to allow quantification of MBF, according to published methods (16,17). To avoid any overlap between the first and second injection of the contrast agent, a pause of 25 s between the prebolus and the main bolus was programmed on the contrast injector (17).

### Quantitative Perfusion Voxel-Wise Analysis

Accurate voxel-based MBF estimation requires respiratory motion correction and myocardial contour delineation. We developed an automated approach based on Refs.(18) and (19), in which respiratory motion is removed using affine image registration by maximization of the joint correlation between consecutive dynamics within an automatically determined ROI. Then, a temporal maximum intensity projection is calculated to serve as a feature image for an automatic contour delineation method based on active contour models (18,19). Signal intensities were then sampled from the corrected data using bilinear interpolation at a grid of 60 angular positions and 10 transmural positions (or layers). The transmural positions were located on chords perpendicular to the myocardial center line (20,21).

Extracted signal intensity (SI) curves were then imported into custom-made in-house software in MATLAB (Mathworks, Natick, MA, version R2010b) which computes perfusion by deconvolving *C*_aif_(*t*), *C*_myo_(*t*), during the first pass of contrast agent in myocardium.

Prior to deconvolution analysis, baseline correction that includes scaling of the signal intensities proportional to coil sensitivity and correcting for an offset to shift the baseline signal to zero was performed. For temporal filtering, a 30th order Hamming window based low pass finite impulse response filter with normalized cutoff frequency of 0.23 (22), was performed on the extracted SI curves. We used 10 time scale (*M* = 10) for exponential bases deconvolution (11) for the representation of impulse response in this study. To render the deconvolution process more stable and reduce the computational burden, ARMA (*Q* = 1, *L* = 2) (23) was chosen for quantification. The length of data used for deconvolution and fit quality assessment is equal to 20 dynamics for all datasets.

### Spatial Resolution Variation

To test the relation between the level of spatial resolution and the accuracy of the deconvolution method, the amount of information loss was evaluated using the following protocol.

**FIG 1:**
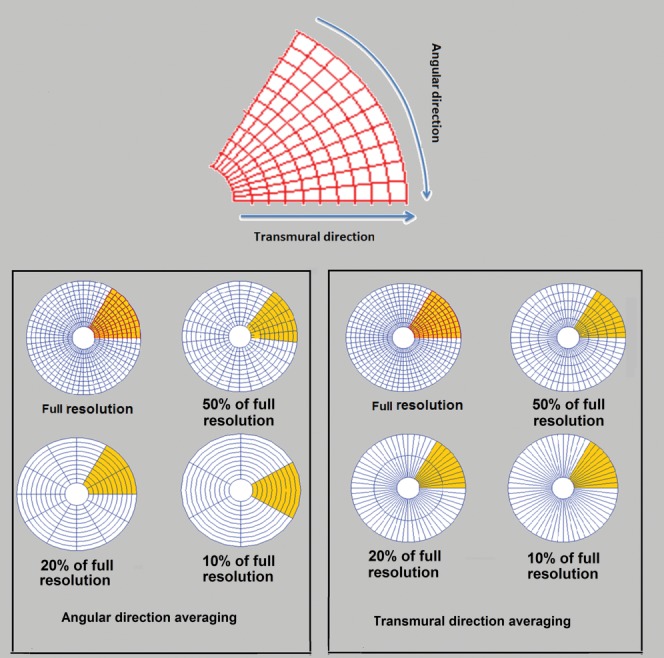
Polar maps illustrating the number of ROI used in one slice of myocardium for different levels of spatial resolution. The polar plot in (a) and (b) shows the number of ROI in one segment while resolution falls down from 100 to 10% in angular direction and transmural direction, respectively.

First, we assigned one voxel to each ROI (600 ROI per slice; in total 1800 ROI per patient) and performed quality of fit analysis on a voxel level (100% resolution). We then incremented the number of voxels assigned to a perfusion ROI in both transmural and angular direction until the resolution fell to 50, 20, and 10% of the original voxel-wise resolution (Fig. 1). Quality of fit analysis was performed. FMI and FRI were estimated at each step of the resolution reduction process. The obtained results from all steps were compared. Contrast to noise ratio (CNR) of the data was calculated at each resolution level. CNR is defined as the ratio of the signal change from baseline to peak of enhancement data, divided by the standard deviation (STD) of the SI curves before contrast. In this study, the ischemic regions in patients group were defined using the full resolution (FR) voxel-wise data and have been extended to the other spatial resolution levels.

Coefficient of variation (CV) was calculated to assess dispersion and variation of each deconvolution method's quality of fit and perfusion estimates due to variation in spatial resolution. It also has been used to compare the sensitivity of methods to direction of spatial resolution reduction.

In this study, FMI was used to evaluate the performance of each deconvolution method at different resolution levels. To compare the accuracy of deconvolution methods at a particular resolution level, FRI has been calculated at all resolution levels.

Mean ± STD of FMI, FRI, and perfusion estimates were calculated for each condition tested to assess and compare the accuracy of the quantification methods. Analysis of variance (ANOVA) was used to compare perfusion estimates and FMI obtained at different resolutions for each deconvolution method. *P*-values were calculated for the null hypothesis that there is no difference between FRI values at different resolution levels, where *P*-values of *P* < 0.05 denoted significant difference.

## RESULTS

**FIG 2:**
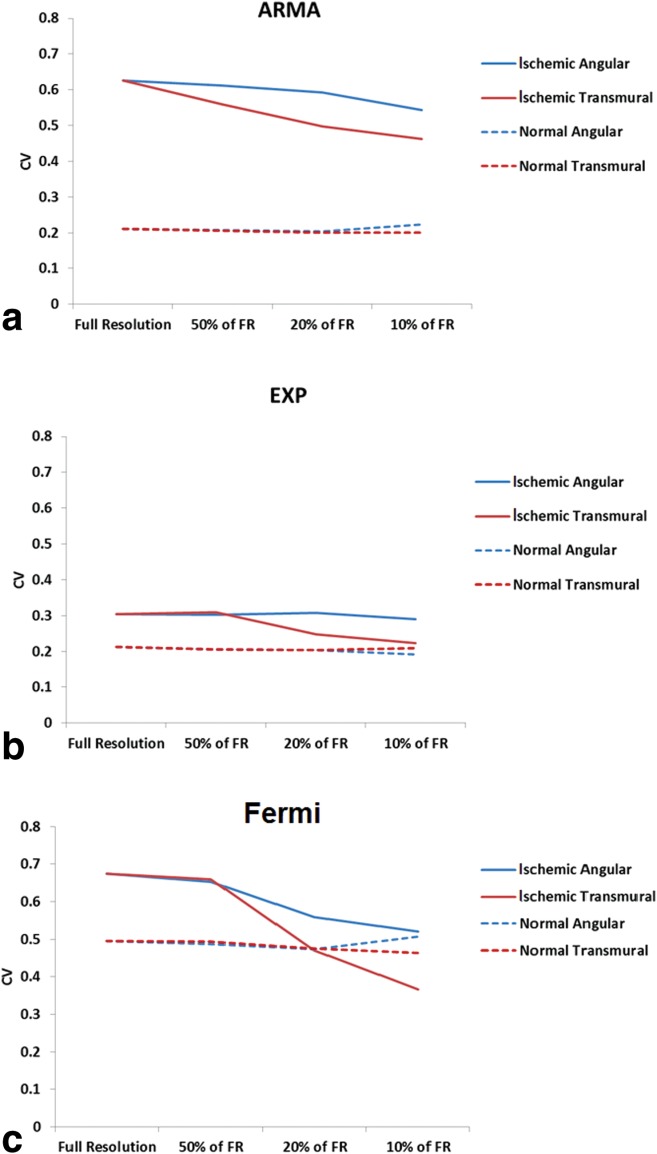
MBF estimates CV for ischemic (solid line) and normal regions (dashed line) in a group of patients is represented as a function of spatial resolution for ARMA, exponential, and Fermi model is represented in (a),(b), and (c), respectively. Differences were observed comparing ischemic regions CV at transmural and angular direction for all three deconvolution methods. This was more evident for Fermi function modeling. For the remote regions, however, the CV remained almost constant for both averaging directions for all methods.

Average perfusion estimates in remote and ischemic regions of myocardium in the group of patients obtained from three deconvolution methods are represented in Table 1. The values are calculated at different resolution levels (i.e., FR, 50, 20, and 10% of FR) and spatial-averaging direction (transmural and angular). Perfusion-estimates CV for ARMA, exponential, and Fermi model is represented in Figure 2. CV was evaluated to represent the loss of information due resolution reduction. Differences were observed comparing ischemic regions CV at transmural and angular direction for all three deconvolution methods. For the ischemic regions, at FR a higher CV was observed as there were more voxels in the ischemic region (selected ROI). CV dropped by reduction in resolution levels due the lower number of voxels and the smaller extent of MBF values. For the remote regions, however, the CV remained almost constant at both averaging directions.

**Table 1:** Average MBF (mL/g/min) in Ischemic and Normal Regions of the Group of Patients at Different Resolution Levels for Both Transmural and Angular Direction of Averaging

		Full	Angular			Transmural		
	
			50%	20%	10%	50%	20%	10%
ARMA	Ischemic	1.28 ± 0.8	1.29 ± 0.79	1.3 ± 0.77	1.29 ± 0.7	1.35 ± 0.74	1.224 ± 0.57	1.15 ± 0.51
	Normal	2.8 ± 0.59	2.8 ± 0.58	2.8 ± 0.57	2.7 ± 0.6	2.83 ± 0.58	2.84 ± 0.57	2.85 ± 0.57
Exp	Ischemic	1.38 ± 0.42	1.39 ± 0.42	1.37 ± 0.42	1.38 ± 0.4	1.39 ± 0.4	1.57 ± 0.4	1.43 ± 0.3
	Normal	2.4 ± 0.51	2.49 ± 0.51	3.1 ± 0.63	2.46 ± 0.47	2.49 ± 0.5	3.1 ± 0.6	2.4 ± 0.5
Fermi	Ischemic	0.86 ± 0.58	1.44 ± 0.94	1.14 ± 0.63	1 ± 0.53	0.88 ± 0.58	1.32 ± 0.62	1.04 ± 0.38
	Normal	2.1 ± 1.04	2.67 ± 1.3	2.68 ± 1.27	1.99 ± 1.01	2.8 ± 1.38	2.69 ± 1.28	2.2 ± 1.02

**FIG 3:**
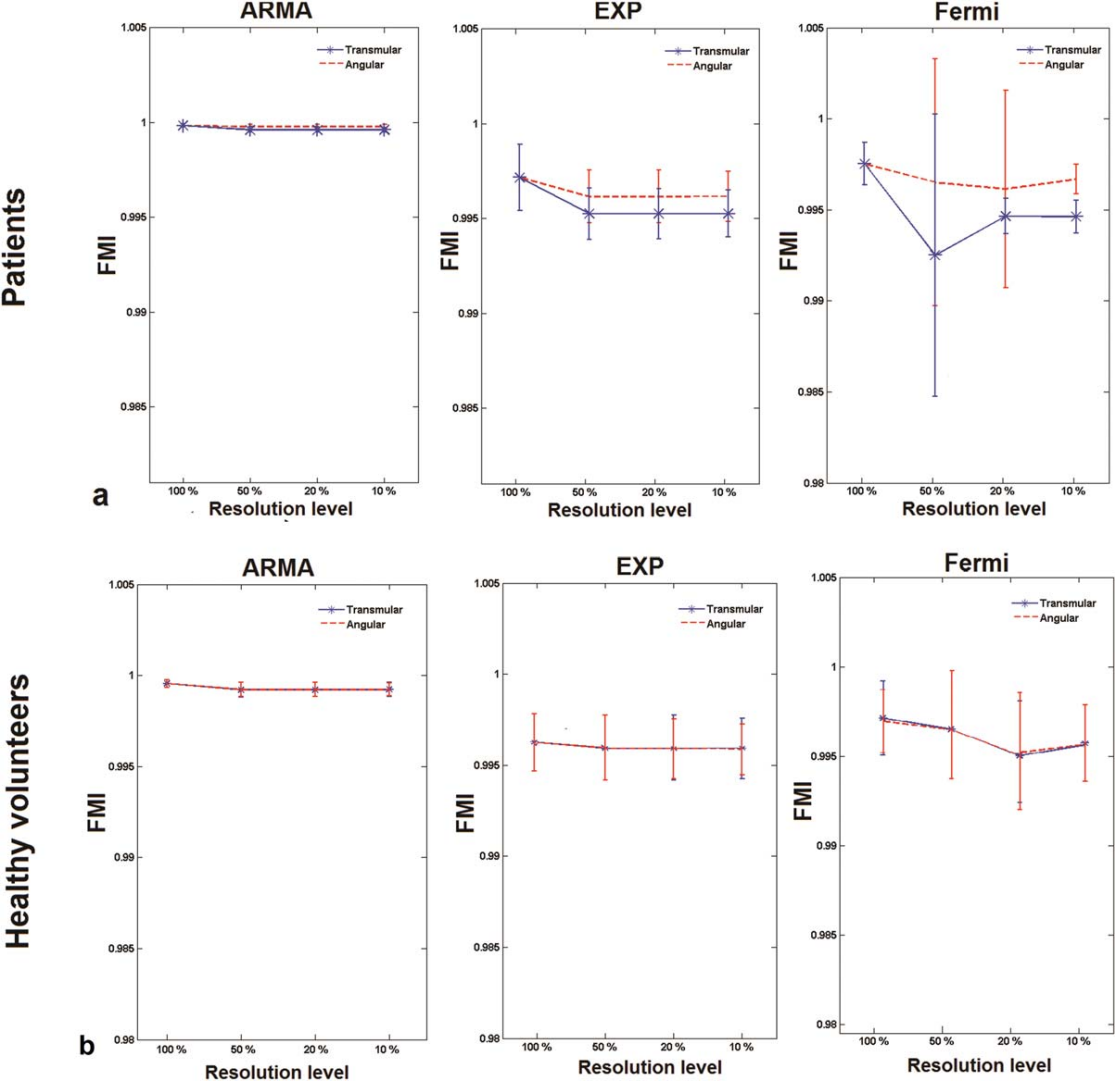
Bars represent mean and STD of FMI at different levels of spatial resolution in (a) a group of patients and (b) a group of healthy volunteers. Solid and dashed lines correspond to reduction of resolution in transmural and angular directions, respectively. Mean FMI value decreased as resolution fell for all methods. This is more obvious for Fermi and exponential methods at transmural direction averaging.

Figure 3 represents FMI mean and STD of deconvolution methods at different resolution levels in the group of patients (Fig. 3a) and healthy volunteers (Fig. 3b). In general for a given set of patient's data, average FMI decreased as the resolution falls down for all deconvolution methods. This decrease was more evident for Fermi and exponential method particularly at transmural direction averaging. The average FMI however remained almost constant in the group of healthy volunteers for all methods in both spatial-averaging directions (Fig. 3b).

This sensitivity of the Fermi and exponential FMI to the direction of spatial averaging is also reflected in the comparison between transmural and angular direction FMI at each resolution level (Table 2). Fermi and exponential showed a significant difference at 50% resolution level (Fermi *P* < 0.001, exponential *P* = 0.0014), whereas no significant differences were observed for ARMA (*P* = 0.081).

**Table 2:** *P*-Values for Differences Between FMI Values of Transmural Direction and Angular Direction Spatial Averaging in a Group of Patients

	50%	20%	10%
ARMA	0.081	0.31	0.64
Exponential	0.0014	0.038	0.041
Fermi	*P* < 0.001	0.003	0.028

*P*-values were calculated for the null hypothesis that there is no difference between FRI values at different resolution levels, where *P*-values of *P* < 0.05 denoted significant difference.

**FIG 4:**
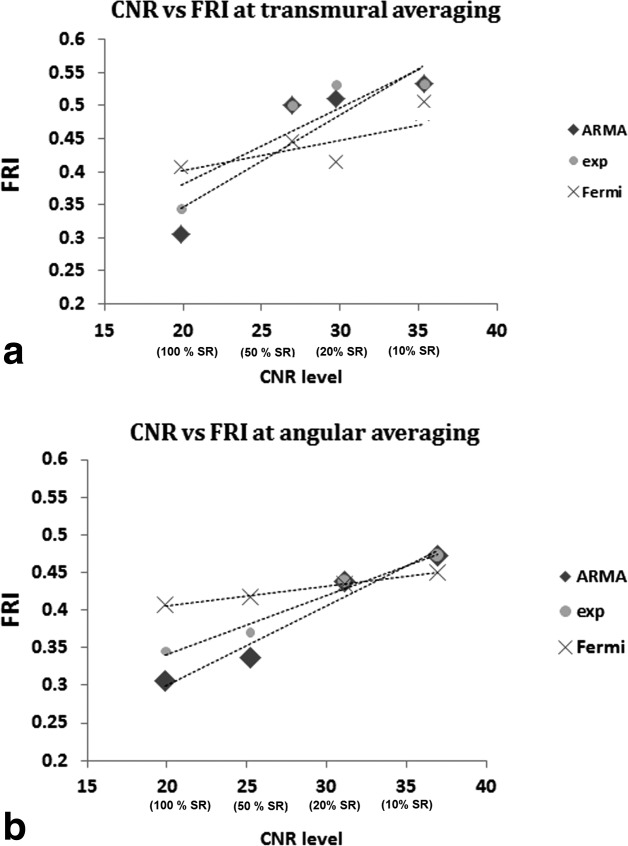
Scatter plot representing the FRI versus CNR level for spatial averaging at (a) transmural and (b) angular direction. CNR level of 20–40 corresponds to FR data to data with 10% spatial resolution (SR), respectively. FRI has increased for all methods in both direction of spatial averaging as CNR improves. Although spatial averaging of the data results in increase of the CNR level and reduction in the error introduced by noise, it will affect the accuracy of quantification by leaving useful information unmodeled. At FR level (CNR = 20) ARMA was the most accurate method among all. At CNR = 35 (segmental analysis), all method had almost similar accuracy.

Comparing the accuracy of deconvolution methods at one particular resolution level, they behaved differently dependent on the resolution level and averaging direction. Figure 4a and b represents scatter plots of the patient group FRI values versus the CNR level (or resolution level) for transmural and angular direction spatial averaging, respectively. At FR, the lowest and highest FRI were belonged to ARMA and Fermi model, respectively (transmural ARMA =0.3, Fermi= 0.41). This indicates a higher amount of modeled information by ARMA model in comparison to Fermi at FR level.

When resolution is reduced by 50% at angular direction ARMA is still the most accurate method. However, Fermi function modeling takes the lead for averaging is in transmural direction. At the CNR level of 35 (10% of FR) all methods had almost similar FRI (average FRI transmural = 0.46, angular = 0.51).

**FIG 5:**
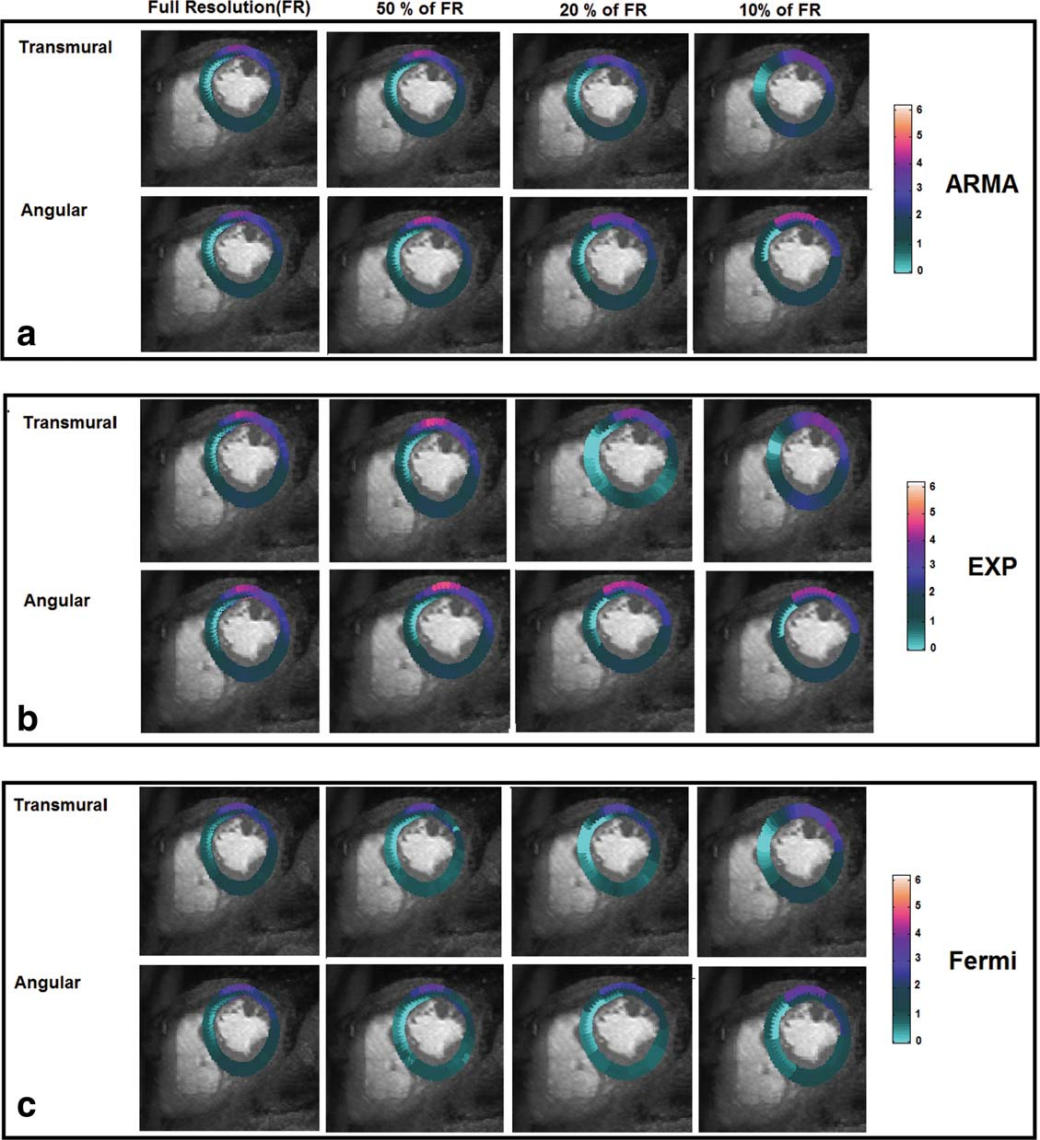
Perfusion maps of a patient with LAD disease for the mid-cavity slice obtained by (a) ARMA, (b) exponential, and (c) Fermi deconvolution. The perfusion maps are represented as function of spatial resolution level and direction of spatial averaging. Perfusion estimates are in mL/g/min. Transmural perfusion variations across the myocardium wall in ischemic regions have been disappeared at 20 and 10% of original resolution in transmural direction averaging for exponential and Fermi methods. However, for angular direction averaging, the endocardium, epicardium, and their transmural perfusion differences are still distinguishable from each other at 20 and 10% resolution levels in ischemic regions.

Perfusion maps of a patient with left anterior descending (LAD) disease as a function of spatial resolution level and direction of spatial averaging for ARMA, exponential, and Fermi model are represented in Figure 5a, b, and c, respectively. Perfusion values are represented in mL/g/min. Note that the transmural perfusion variations across the myocardium wall in ischemic regions have disappeared at 20 and 10% of original resolution in transmural direction averaging for exponential and Fermi methods. However, for angular direction averaging the endocardium, epicardium, and their transmural perfusion differences are still distinguishable from each other at 20 and 10% resolution levels in ischemic regions.

*P*-values represented in Table 3 are the result of performed ANOVA test on the perfusion estimates at different resolution levels on a group of patients. There was a significant difference (*P* < 0.05) between FR MBF and MBF obtained from same dataset at 20 and 10% of FR level in transmural direction. For spatial averaging, at angular direction significant difference was found between the perfusion estimate at a FR and the one from 10% of FR for ARMA and exponential. However, Fermi showed to be sensitive to spatial averaging in angular direction at 20% of FR level as well (*P* < 0.001).

**Table 3:** *P*-Values for Differences Between MBF Estimates at Different Spatial Resolution Levels of Transmural Direction and Angular Direction Spatial Averaging in a Group of Patients (FR = Full Resolution)

		FR versus 50% of FR	FR versus 20% of FR	FR versus 10% of FR
ARMA	Transmural	0.58	*P* < 0.001	*P* < 0.001
	Angular	1	0.405	0.003
EXP	Transmural	1	0.015	*P* < 0.001
	Angular	1	0.703	*P* < 0.001
Fermi	Transmural	8 E −02	*P* < 0.001	*P* < 0.001
	Angular	4 E −01	*P* < 0.001	*P* < 0.001

*P*-values were calculated for the null hypothesis that there is no difference between FRI values at different resolution levels, where *P*-values of *P* < 0.05 denoted significant difference.

## DISCUSSION

Myocardial ischemia affects the subendocardial layers of the LV myocardium earlier and more severely than the outer layers (24). The high spatial resolution of myocardial perfusion CMR allows the visualization of subendocardial ischemia (21).

Postprocessing of these high-resolution perfusion CMR data can be used to quantify the imbalances between subendocardial and subepicardial myocardial perfusion. It allows for preserving important spatial information and is a superior sensitive method for subendocardial ischemia detection, similarly to visual assessment.

However, high-resolution quantification suffers from low SNR of the SI curves, which is one of the major causes of inaccuracies in estimation of MBF.

In the present study, we have investigated the effect of spatial averaging (data resolution level) on the accuracy of the deconvolution methods. We have used a quality assessment method introduced by Balvay et al. (12) to estimate the amount of modeled data (FMI) remaining unmodeled-information (FRI) at each spatial resolution level.

### Our Data Confirms the Following Findings

1. While spatial averaging of the data results in increase of the CNR level and reduction in the error introduced by noise, it affects the accuracy of quantification by leaving useful information unmodeled at low-resolution levels.
2. This information loss is more severe when the spatial resolution reduction is in transmural direction. This is a particularly interesting finding that agrees with the pathophysiology of myocardial ischemia and with the preferential subendocardial localization of myocardial ischemia (21). In fact, subendocardial ischemia is more likely to be missed as result of spatial averaging performed in the transmural direction compared with the radial direction. In this case, the SI form subendocardial ischemic segments is averaged with the mid and epicardial layers, which have relatively preserved perfusion. In case of radial averaging instead, the signal from ischemic subendocardial layers is averaged within the same layer, thus limiting the detrimental effects of spatial averaging to the border between ischemic and normally perfused coronary artery territories. Moreover, this also potentially explains why no significant differences between transmural and radial averaging were observed in normal volunteers. The absence of perfusion abnormalities in healthy volunteer's results in the lack of effect of spatial averaging on the amount of modeled information, as there is no additional information available at transmural directional in subendocardial layer.
3. At high spatial resolution, ARMA model was able to model the highest amount of information in the tissue compared to other methods (i.e., low FRI value, FRI = 0.305, and higher FMI ≈ 1). It also showed to be the most robust method to changes in level of spatial resolution at both angular and transmural direction averaging.
4. At low spatial resolution levels, the amount of modeled information was almost equal for all four methods (similar FRI).
5. No changes concerning mean MBF values at all resolution levels were observed in both ischemic and normal regions for ARMA and exponential methods. Similarly, no perceptible drop was observed for the estimated FMI values for ARMA at all resolution levels and exponential after 50% reduction in resolution level.
6. The average FMI value for Fermi model dropped significantly by reduction in resolution level. Also evident differences were observed between mean MBF values obtained by Fermi at different resolution levels. These suggest a greater sensitivity of Fermi model to changes in level of spatial resolution especially in the transmural directions.
7. On average for all quantification methods, based on actual perfusion and FMI measurement, at least 50% of full spatial resolution (i.e., voxel size of 2 × 2 mm^2^) is necessary to obtain reliable perfusion estimates and avoid loss of physiological information due to spatial averaging. Reducing the spatial resolution level more than 50% at transmural and 20% at angular direction will result in losing information about transmural perfusion differences.

In general, a greater difference between ischemic and normal regions estimated MBF values was observed compared to the differences in estimated MBF due to resolution level in either of the regions for all methods. This along with reduction in FMI and CV values as a result of reducing resolution in ischemic regions suggests that spatial resolution reduction affects the evaluation of the extension and spatial profile of the ischemic regions rather than the estimated MBF range. Here, we have assumed that the noise which has contaminated the data is an additive white Gaussian noise with zero-mean and normal distribution. However, after filtering the data using a low pass filter noise cannot be approximated as white noise anymore. Therefore, this assumption will have an effect on FRI and FMI estimation. Another factor which might affect the efficiency of fit quality assessment is the number of time points (length of the data) used for analysis. For the current study, we have used 20 dynamics for all datasets.

MR images which are used in this study are chosen based on a minimum SNR and quality level criteria for an accurate quantification (25). Temporal filtering also has been applied to these images to improve the quality and reduces the noise prior to quantification process. Further work is required to investigate the relation between FMI, temporal filtering level and the image quality of different MR acquisition techniques at high-resolution level. Effects of improvement in the MRI acquisition techniques for a better resolution level and SNR on the quantification accuracy remain to be seen.

## CONCLUSIONS

In this study, we have demonstrated that decreasing the resolution level to improve the SNR of the data will result in losing important physiological information which can be possibly used for arriving at a clinical diagnosis. Higher resolution of the CMR images will result in better detection of subendocardial ischemia and potentially more accurate and sensitive in measuring the amount of ischemia.

All deconvolution methods were more sensitive to spatial resolution averaging in the transmural direction. ARMA model showed to the least sensitive methods to both changes in spatial resolution levels and direction of averaging.
